# Assimilable Organic Carbon (AOC) in Soil Water Extracts Using *Vibrio harveyi* BB721 and Its Implication for Microbial Biomass

**DOI:** 10.1371/journal.pone.0028519

**Published:** 2012-05-04

**Authors:** Jincai Ma, A. Mark Ibekwe, Menu Leddy, Ching-Hong Yang, David E. Crowley

**Affiliations:** 1 United States Salinity Laboratory, United States Department of Agriculture-Agriculture Research Service, Riverside, California, United States of America; 2 Department of Environmental Sciences, University of California Riverside, Riverside, California, United States of America; 3 Orange County Water District, Fountain Valley, California, United States of America; 4 Department of Biological Sciences, University of Wisconsin, Milwaukee, Wisconsin, United States of America; Argonne National Laboratory, United States of America

## Abstract

Assimilable organic carbon (AOC) is commonly used to measure the growth potential of microorganisms in water, but has not yet been investigated for measuring microbial growth potential in soils. In this study, a simple, rapid, and non-growth based assay to determine AOC in soil was developed using a naturally occurring luminous strain *Vibrio harveyi* BB721 to determine the fraction of low molecular weight organic carbon in soil water extract. Calibration of the assay was achieved by measuring the luminescence intensity of starved *V. harveyi* BB721 cells in the late exponential phase with a concentration range from 0 to 800 µg l^−1^ glucose (equivalent to 0–16.0 mg glucose C kg^−1^ soil) with the detection limit of 10 µg l^−1^ equivalent to 0.20 mg glucose C kg^−1^ soil. Results showed that bioluminescence was proportional to the concentration of glucose added to soil. The luminescence intensity of the cells was highly pH dependent and the optimal pH was about 7.0. The average AOC concentration in 32 soils tested was 2.9±2.2 mg glucose C kg^−1^. Our data showed that AOC levels in soil water extracts were significantly correlated (*P*<0.05) with microbial biomass determined as microbial biomass carbon, indicating that the AOC concentrations determined by the method developed might be a good indicator of soil microbial biomass. Our findings provide a new approach that may be used to determine AOC in environmental samples using a non-growth bioluminescence based assay. Understanding the levels of AOC in soil water extract provides new insights into our ability to estimate the most available carbon pool to bacteria in soil that may be easily assimilated into cells for many metabolic processes and suggest possible the links between AOC, microbial regrowth potential, and microbial biomass in soils.

## Introduction

Availability of carbon has been assumed to be the primary determinant factor for bacterial growth in both water and soil, although there are reports of limitation by other nutrients, e.g. nitrogen and phosphorus [1]. In aqueous media, the most common methods to estimate carbon availability are measurements of biodegradable dissolved organic carbon (BDOC), biological oxygen demand (BOD), and assimilable organic carbon (AOC). BDOC is the fraction of the dissolved organic carbon (DOC) which can be metabolized by bacteria within a few days to a few months. BDOC measures the change in dissolved organic carbon (DOC) in a water sample after incubation with microorganisms for a period of time. [2]. AOC is the most bioavailable fraction of BDOC since it can be readily assimilated by microorganisms for growth [3], . Likewise in soils, carbon is generally fractionated into pools with different degrees of recalcitrance, primarily to determine carbon turnover rates under different environmental conditions and soil management [5]. Currently, there are no defined methods for describing the equivalent of AOC for carbon in soil water extracts. Nonetheless, such methods would be very useful for estimating the amounts of carbon that are immediately available to support microbial growth and to determine how the amount of readily assimilable carbon in soils and other environmental samples are influenced by a host of biological and environmental variables.

The composition of AOC in water consists of a broad spectrum of organic compounds, including sugars, organic acids, amino acids, and peptides, whose molecular weights are typically less than 1,000 Daltons [6]. Although AOC accounts for about 0.1–9% [7] of the total organic carbon (TOC) in water, it is an important indicator for evaluating the regrowth potential of heterotrophic microbes [8], [9]. A recent study by Vital et al [10] showed that AOC is one of the major determinants controlling the growth of the selected pathogenic bacteria (*Escherichia coli* O157, *Vibrio cholerae*, and *Pseudomonas aeruginosa*) in water. Extending the AOC concept to soil is useful as it may provide an indication of the potential growth and survival of pathogens, and allocthonous microorganisms that are introduced into the soil with amendments, or other sources. It is also of interest to examine the extent to which AOC concentrations under steady state conditions are correlated with total organic carbon and the soil microbial biomass, and the extent to which it is affected by soil management, *e.g.* tillage, wet dry cycles, flooding, or other environmental factors.

On the average, AOC concentrations in water range between ten to several hundred µg C l^−1^. Typically, limited growth of bacteria is expected when AOC is less than 20 µg C l^−1^; however, significant growth of coliform bacteria has been observed when AOC concentrations exceed 50 µg C l^−1^ [11], [12]. Compared to AOC in water, AOC levels in soils are still largely unknown due to lack of methods for measuring this carbon pool, but it is hypothesized that the AOC in soil might be established by measuring the AOC in water-soluble organic carbon extracted from soil samples using well established protocol [13]. It is well known that the additions of organic carbon to soil can increase soil microbial activity [14], alter microbial community compositions [15], [16], and increase the cell densities of indigenous bacteria and fungi [17].

Conventional methods for AOC in water are growth based assays. The first AOC test was originally developed by van der Kooiji and colleagues [7], improved by Kaplan et al. [18], LeChevallier et al. [19], and later adopted as a standard method [20]. In these studies water samples were inoculated either with pure cultures including *Pseudomonas fluorescens* P-17 and *Spirillum* sp. strain NOX, which have been well characterized to show substrate utilization, or with a natural microbial community, which might help broaden the range of substrates utilized by the bacteria in the community [21]. After the test samples were incubated for several days until the AOC has been fully converted into microbial biomass. The AOC concentrations in water samples were calculated by relating the equivalent yields of bacteria that were produced on defined substrates (acetate or oxalate carbon) to generate a standard curve. According to the operational definition of AOC, most of the previous AOC assays were growth-based, therefore, classical AOC tests are time-consuming and labor-intensive. With water samples, it takes up to one week to achieve the full yield of microbial biomass after inoculation with the test microorganisms. The methods also involve expensive procedures, e.g. flow cytometry, in quantification of the resultant microbial biomass [21]. To simplify this method, the widely used AOC test bacteria, P-17 and NOX, were genetically modified to produce bioluminescence by integrating *luxCDABE* operon into their genomic DNA, and later a bioluminescence-based test using both artificially constructed luminous strains for AOC in water was developed [22], [23]. However, days of incubation are still necessary for the genetically engineered bacteria to reach optimal growth. As a result, the bioluminescence intensity of the water sample in relation to that of the standard sample supplemented with a known AOC concentration can be used to quantify microbial biomass. Recently, an AOC assay for salt water samples using naturally occurring luminous strain *Vibrio harveyi* was developed [24]. However, this assay was still growth based. In the current study, we have built upon the work of Weinrich et al. [24], and extend the assay to AOC determination in soil water extracts. In our assay glucose was used as a carbon source because it is a simple organic compound that is quickly transported by the cell and quickly broken down. In addition, more complex organic compounds are generally catabolized by the cells into simpler compounds such as glucose. Therefore, both glucose and acetate were selected as standards because they will most likely be present in the sample or broken down into acetate/glucose by the cell. Also, a non-growth based assay will inherently not measure the same organic compounds that a growth-based assay will, because the growth-based assay has had the time to utilize the simple organic compounds for the organism's initial growth and then utilize the more recalcitrant compounds for further growth. Therefore, our assay can be used for quick monitoring of the fraction of AOC that can be easily utilized in soil water extract.

The new assay involves the development of a non-growth bioluminescence based assay for soil AOC measurements using starved cells of the naturally occurring luminous microorganism, *Vibrio harveyi* BB721 [25]. *V. harveyi* is a Gram-negative, bioluminescent, rod-shaped, motile, facultative anaerobic, halophilic marine bacterium capable of fermentative and respiratory metabolism. Recently, this strain has successfully been used to determine AOC in salt water [24]. The objectives of this study were (1) to develop a method for estimating AOC in soil water extract that is rapid, simple, and reproducible with an indicator bacterium that produces bioluminescence in response to AOC levels in soil water extracts; (2) to correlate AOC levels in soil water extracts with microbial biomass as indicated by microbial biomass carbon (MBC).

## Materials and Methods

### Ethics Statement

*Vibrio harveyi* BB721 (ATCC 700106) was obtained from ATCC. No specific permits were required for the described field studies. This is because no studies were conducted on site in any of the locations and soil samples were collected once. Some of the locations used for sampling are not privately-owned or protected and the field studies did not involve endangered or protected species.

### Bacterial strain and growth conditions

*Vibrio harveyi* BB721 (ATCC 700106) was chosen because the strain displays constitutive luminescence, and the amount of light produced is directly proportional to biomass, which can be correlated to AOC in water [24]. The culture stock was stored at −80°C. One glass bead from the storage tube was aseptically transferred into a glass culture tube containing 4.0 ml BOSS medium (pH, 7.2) [26], briefly vortexed to release the cells into the liquid phase, and then 500 µl of the cell suspension was transferred into another culture tube containing 4.0 ml BOSS medium. The cells were incubated at 27°C on a rotary shaker (250 rpm) for about 16 h and harvested by centrifugation at 4000 rpm. The cell pellet was washed 3 times with 1.0 ml diluted (1∶7) MOPS buffer (0.20 M 3-morpholinopropane-1-sulfonic acid and 3.0 M sodium chloride in Milli-Q water, pH, 7.0) then resuspended in the same original volume of diluted MOPS buffer for subsequent experiments.

### Carbon free materials

Carbon free water (Milli-Q water) was used in all the experiments except where otherwise stated. Carbon free glassware was prepared according to the method described previously [20]. In brief, glassware was washed with detergent, rinsed with tap water and deionized water three times, then soaked in 0.2 M HCl overnight. The HCl-treated glassware was then rinsed with Milli-Q water and air dried. Finally, the glassware was covered with aluminum foil and baked at 550°C in a muffle furnace for at least 4 h. Teflon coated screw caps were washed using 0.2 M HCl, immersed into hot (60°C) sodium persulphate solution (10%) for 1 h, rinsed 3 times with deionized water and Milli-Q water, respectively, and then air dried.

### Effect of pH and starvation on the luminescence of *V. harvayi* BB721

A series of phosphate buffer (10 mM) containing three M NaCl with different pH (4.0–8.0) was made by adding 0.1 M HCl or 0.1 M NaOH. Twenty microliters of the cell suspension was transferred to saline water, gently mixed, and incubated in triplicate for 30 min under room temperature (23±1°C). Following the incubation, the Promega Steady-Glo protocol (Promega, Carlsbad, CA) and a luminometer (Turner Biosystem, CA) were used to measure luminescence. For the cell starvation study, the luminescence of the cells in diluted MOPS buffer was measured in triplicate periodically after washing. The normalized luminescence readings were then plotted against the elapsed time.

### Detection limit, effect of cell concentrations and low molecular weight organic carbon molecules

In order to calculate the detection limit of the method, eight blank samples (Milli-Q water) were treated as regular samples to get the background signal and the standard deviation was then calculated. It was assumed that 3 times the standard deviation was the threshold signal corresponding to the lowest AOC concentration that can be calculated from the calibration curve. The calculated detection limit was 10 µg l^−1^ (0.2 mg glucose C kg^−1^ soil). For optimization of linear range and initial cell concentration, starved cells were added to a series of diluted MOPS buffer containing glucose. The concentration of glucose ranged from 0 to 5000 C µg l^−1^. The luminescence intensities were measured after 30, 60, 90, and 120 min incubation at room temperature (23±1°C). Additionally, the effect of initial cell concentrations on luminescence was also investigated by adjusting the volume of cell suspension inoculated into the diluted MOPS buffer containing glucose as a carbon source. Luminescence of BB721 in response to 10 low molecular weight organic carbon sources was also assayed. Those organic carbon sources include acetate, arabinose, citrate, formate, fructose, glucose, lactate, mannose, oxalate, and sorbitol. All chemicals were reagent grade from Sigma-Aldrich, MO.

### Soil collection and AOC determination

Soil samples were collected from 32 soils from CA and AZ. Each sample was a composite of several individual surface soil cores (0–15 cm in depth) to insure the representativeness of the soil samples. Sampling sites were chosen in major fresh produce growing areas in California and Arizona. Bagged samples were taken to the laboratory in ice boxes. Vegetations, roots and stones were removed and the soil sieved using a <2 mm sieve. A sub-sample was freeze-dried, and water soluble organic carbon (WSOC) extraction was conducted. Another sub-sample was air dried for physical and chemical analyses. All samples were characterized for clay, silt, and sand content, water content, water holding capacity (WHC), total organic carbon (OC), and total nitrogen (T-N) according to standard methods [27]. Soil MBC was extracted by the chloroform-fumigation-extraction method [28]. To determine the recovery of the glucose added into the soils, 25 gram of soil were spiked with 10 mg kg^−1^ of glucose and the soil extract was analyzed for glucose following the AOC determination procedure described below.

For AOC determination from soil water extract, 10 g of frozen soil was mixed with 20 ml of Milli-Q water, and subjected to horizontal shaking at room temperature for 30 min [13]. The soil mixture was then centrifuged for 10 min at 5000 rpm at 4°C. The supernatant was filtered through a 0.45 µm nylon filter (Millipore, CA), and stored at 4°C until analyzed for WSOC and AOC. For the AOC determination, *V. harveyi* cells were grown in BOSS medium for 16 h (late exponential phase, OD_600 nm_, ∼1.8), centrifuged at 14000 rpm for 30 sec, washed twice using diluted MOPS buffer, and then starved in the same buffer for 30 min. Glucose carbon stock solution (5000 mg l^−1^) was prepared by dissolving 0.625 g of glucose (Sigma-Aldrich, St Louis, MO) in 50 ml of Milli-Q water. This stock solution was then subjected to 10-fold serial dilution to get 5.0 mg l^−1^ working solution. The AOC standard series was prepared by diluting the 5.0 mg l^−1^ glucose solution into the tube containing diluted MOPS buffer. For sample preparation, 100 µl of soil water extract, 125 µl MOPS buffer, and Milli-Q water were added into the reaction vial (1.5 ml Eppendorf centrifuge tube) to make a final volume of 1000 µl. In all the reaction vials, including standard series and samples, 20 µl of starved cell suspension was added and the sample tubes were inverted 6 times, and left on bench top under room temperature for 30 min without shaking. The bioluminescence was then measured using a luminometer as described above. The calibration curve was constructed by plotting the standard glucose carbon concentration (0–800 µg C l^−1^) against intensity of bioluminescence. The AOC concentration in the soil water extract was calculated from the calibration curve, and finally the AOC concentration in soil (mg glucose C kg^−1^) was obtained using the following equation:
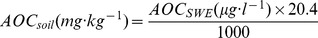
where AOC_soil_ is AOC level in soil and AOC_SWE_ is the AOC fraction in soil water extract, and 20.4 is the dilution factor.

### Statistical analysis

Linear regression analysis of soil AOC, WSOC, , and MBC was performed, and Pearson correlation coefficients between soil organic fractions and soil properties were determined using the SPSS 16.0 software package (Chicago, IL).

## Results

### Effects of pH and cell starvation on cell bioluminescence

**Figure 1:**
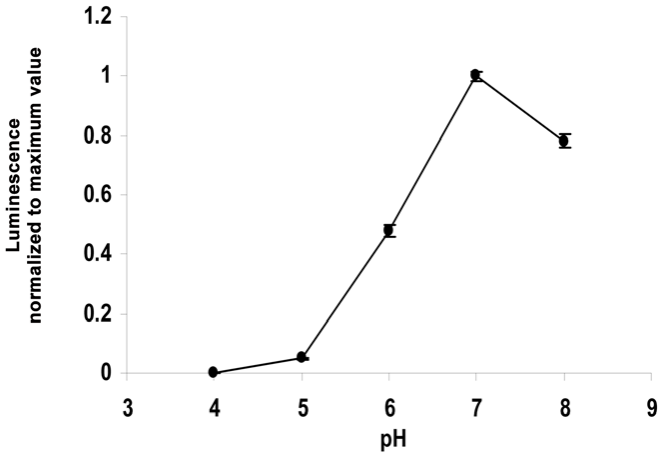
Effect of pH on Luminescence of *V. harveyi*. The data represent the average of triplicate soil measurements.

**Figure 2:**
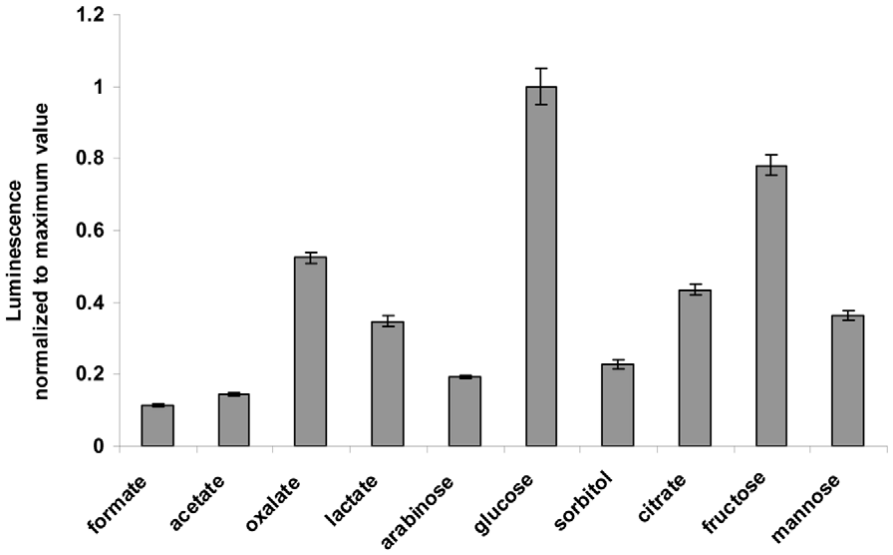
Luminescence strength in response to low molecular weight organic carbon sources. The luminescence of the starved cell in 10 mM MOPS buffer (pH, 7.0) was measured after 30 min of incubation without shaking. The organic carbon source was added to a final carbon concentration of 100 µg l^−1^. The data represent the average of triplicate soil measurements.

The bioluminescence of cells was strongly affected by the pH of the medium (Fig. 1). Bioluminescence was almost completely inhibited when pH was less than 4, and increased in a linear fashion with increase in pH to a maximum value that was observed at a pH of approximately 7.0. The pH of the buffer was thus adjusted to 7.0 during the subsequent experiments unless otherwise stated. Cell starvation after harvest and washing at the late exponential growth phase were examined by monitoring bioluminescence over time in ten low molecular weight organic carbon substrates (Fig. 2). Results showed that once the washed cells were placed into the buffer solution, bioluminescence declined sharply within the first 5 min, followed by a slow decrease for about 30 min (data not shown). Thereafter, the cells produced a relatively steady luminescence for up to 75 min. The results from Fig. 2 show that the maximum luminescence signal was observed when BB721 was added to media containing glucose. Fructose and oxalate generated 78% and 52% of the luminescence intensity produced by glucose. Citrate and mannose were also good carbon sources for the cell to produce light, and the efficiencies were 44% and 36% of glucose. Other carbon sources tested had relatively low efficiency in stimulating the production of light in BB721, ranging from 11% to 23% of that of glucose. Therefore, glucose was chosen as the standard carbon source to construct the calibration curve as it allowed the greatest sensitivity for detecting low amounts of carbon, expressed as glucose equivalents in µg C l^−1^.

### Effect of incubation time and inoculum size on bioluminescence

**Figure 3:**
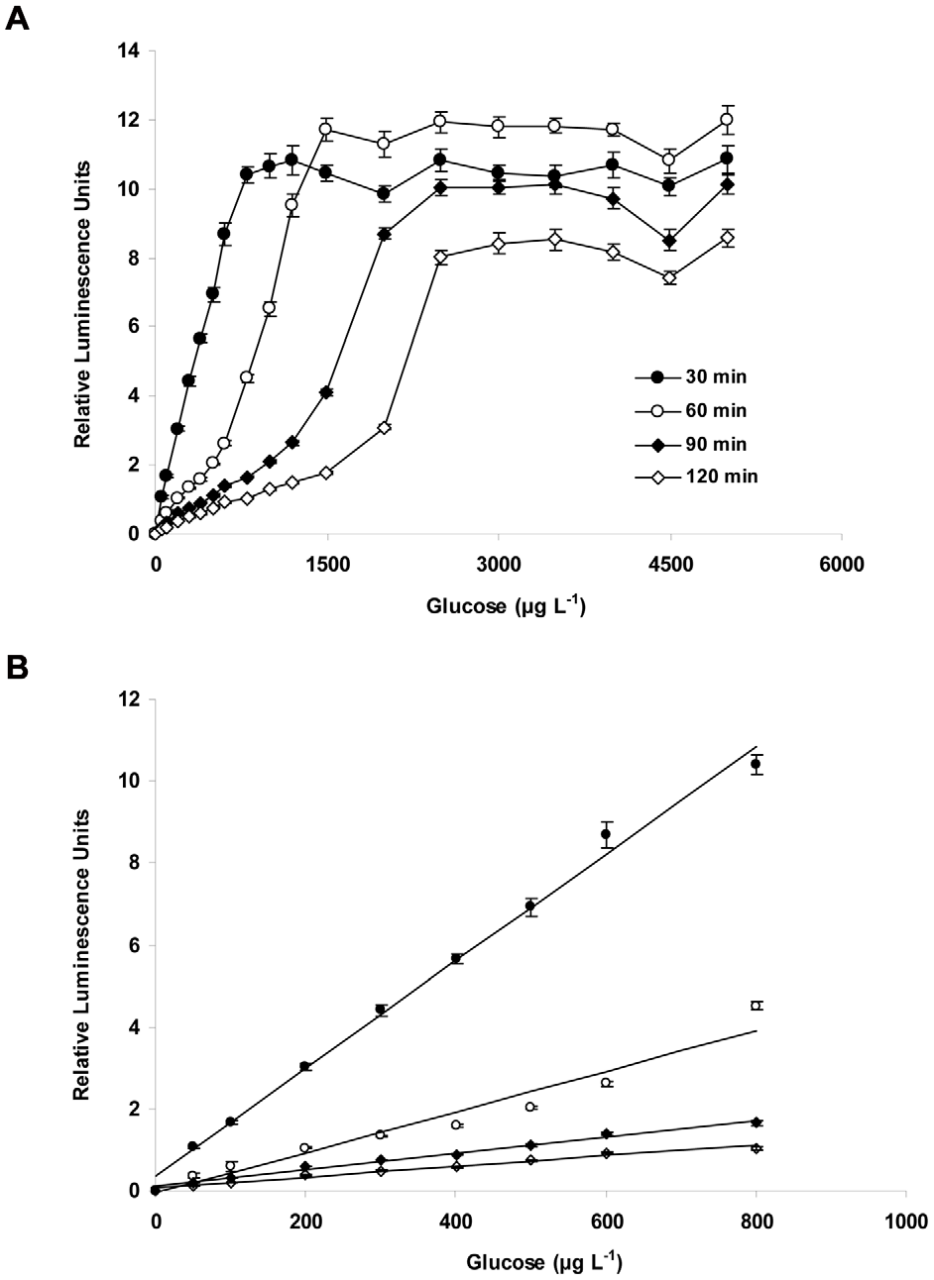
Relative luminescence strength of *V. harveyi* in response to different glucose concentration and incubation time (3A), and linear range (3B). Cell suspension (optical density at 600 nm about 1.5) added was 20 µl, incubation time varied from 30 min to 120 min. The data represent the average of triplicate soil measurements.

The production of luminescence by the starved cells in the presence of glucose was tested at concentrations ranging from 0 to 5000 µg C l^−1^ and for different incubation times (30 min to 120 min) (Fig. 3A&B). For a given incubation time, the intensity of luminescence increased with increases of glucose concentration, however, the signal became saturated when glucose concentrations exceeded 1000 to 1500 µg l^−1^ over the range of incubation times that were tested (Fig. 3A). When inoculum having an OD_600 nm_ of 1.5 was introduced to test solutions at different volumes ranging from 5 to 20 µl ml^−1^, low inoculum volumes required increased incubation time, with a concomitant decreased in linear range and sensitivity (Table 1). On the other hand, for fix incubation time, the increase of inoculum quantity led to improvement of the linear range and sensitivity. Overall, the best linear range and sensitivity was obtained when inoculum size was 20 µl and incubation time was 30 min (Fig. 3B). Therefore, in subsequent experiments, 20 µl suspensions of starved cell having an OD_600 nm_≈1.5 were used to inoculate 1.0 ml of the assay mixture, with an incubation time of 30 min before measuring bioluminescence.

**Table 1:** Effect of initial cell concentration and incubation time on sensitivity of the method.

	Incubation time (min)	Cell suspension volume (OD_600 nm_∼1.5) added		
		5 µl	10 µl	20 µl
linear range (µg l^−1^ glucose C)		0–400	0–500	0–800
	30	0.0083	0.0114	0.0135
	60	0.0071	0.0059	0.0052
	90	0.0037	0.0013	0.0022
	120	0.0016	0.0007	0.0014

### AOC concentration in soil water extract and correlation with MBC

A total of 32 soils (16 organic, 16 conventional), were collected from three major fresh produce-growing areas in California and Arizona. Soil prosperities are listed on Table 2. Yuma and Imperial Valley shared similar weather conditions including MAT, MAP, due to the closeness of the two locations in comparison to Salinas Valley (Table 2). Soil pH was between 6.7 and 8.0. The average AOC content (Table 2) in the soil was 2.92 mg kg^−1^ with the highest measured value of 8.80 mg kg^−1^ in a soil having a clay texture (AZ-O2 sample), and the lowest value of 0.66 mg kg^−1^ was measured in a sandy loam soil (IM-O3 sample). The average standard deviation for triplicate measurements of soil samples was 2.62 mg kg^−1^. For samples that were spiked with known concentrations of glucose, the average glucose carbon recovery was 93±12% of that measured for aqueous samples used to prepare the standard curves.

**Table 2:** Soil properties.

Soil ID	coordination	MAT (°C)	MAP (mm)	soil management	soil texture	pH	EC (dS m^−1^)	WHC (%)	Silt (%)	Clay (%)	T-N (%)	OC (%)	WSOC (mg kg^−1^)	AOC (mg kg^−1^)	AOC/WSOC (%)
AZ-C1	LAT: 32.69°N, LON: 114.61°W.	23.9	76.5	Conv	clay	7.9	1.945	47.5	39.9	43.9	0.07	1.81	54.2	1.66	3.07
AZ-C2				Conv	clay	8.0	2.170	47.9	38.5	42.8	0.06	1.78	37.1	1.73	4.65
AZ-C3				Conv	silty clay	7.9	1.929	51.2	38.5	44.0	0.10	1.76	47.2	1.50	3.18
AZ-C4				Conv	silty clay	7.9	1.836	47.7	42.8	42.2	0.12	1.86	57.0	2.41	4.24
AZ-C5				Conv	clay loam	8.0	1.874	49.4	40.3	38.5	0.05	1.77	52.0	1.99	3.82
AZ-C6				Conv	clay loam	8.0	2.160	44.0	36.5	39.8	0.07	1.91	48.7	1.56	3.20
AZ-O1				Org	clay	7.9	1.182	51.2	38.5	44.0	0.07	1.91	133.8	6.51	4.86
AZ-O2				Org	clay	8.0	1.238	47.8	38.2	44.3	0.06	1.95	118.3	8.83	7.46
AZ-O3				Org	silty clay	7.9	1.213	47.4	40.8	41.7	0.06	1.91	144.9	7.27	5.02
AZ-O4				Org	silty clay	8.0	1.179	46.6	40.8	41.7	0.06	1.79	100.5	7.76	7.73
AZ-O5				Org	clay loam	8.0	1.287	53.4	42.9	39.6	0.07	1.93	72.8	1.60	2.20
AZ-O6				Org	clay loam	8.0	1.228	42.1	42.9	39.6	0.06	1.88	85.4	4.72	5.52
IM-C1	LAT: 32.85°N, LON: 115.49°W.	22.8	74.2	Conv	clay	7.7	1.171	47.2	39.1	41.0	0.06	1.77	43.7	0.84	1.91
IM-C2				Conv	clay	7.5	3.600	45.4	26.3	43.7	0.09	2.05	65.0	3.45	5.31
IM-C3				Conv	sandy loam	7.7	0.802	38.8	16.6	18.4	0.04	1.26	44.4	1.16	2.62
IM-C4				Conv	sandy loam	7.8	1.460	34.6	20.8	14.2	0.04	1.50	67.7	3.23	4.78
IM-C5				Conv	clay loam	7.8	0.981	43.2	42.1	35.5	0.04	1.96	75.2	1.20	1.60
IM-C6				Conv	clay loam	7.6	1.456	30.8	42.1	35.5	0.06	2.20	51.3	1.12	2.18
IM-O1				Org	clay	7.7	1.425	58.6	26.4	56.2	0.08	2.06	102.9	3.08	3.00
IM-O2				Org	clay	7.7	3.030	43.8	34.9	47.7	0.11	2.14	43.3	1.31	3.03
IM-O3				Org	sandy loam	8.1	0.908	30.4	8.3	16.7	0.05	1.21	68.2	0.66	0.97
IM-O4				Org	sandy loam	7.8	1.102	36.5	22.0	16.8	0.06	1.59	72.5	1.93	2.67
IM-O5				Org	clay loam	7.7	2.130	39.9	33.6	36.5	0.11	2.10	49.5	1.91	3.86
IM-O6				Org	clay loam	7.7	1.708	39.9	38.2	29.4	0.07	1.97	55.3	0.89	1.61
SA-C1	LAT: 36.69°N, LON: 121.58°W.	14.4	328	Conv	sandy loam	7.4	0.638	21.4	13.1	8.8	0.13	1.28	52.1	1.40	2.68
SA-C2				Conv	loamy sand	6.8	0.177	19.4	13.0	6.3	0.03	0.51	72.7	1.69	2.33
SA-C3				Conv	loam	7.8	0.371	31.0	28.8	15.5	0.09	1.13	65.1	1.37	2.11
SA-C4				Conv	clay loam	8.1	0.509	60.3	44.6	33.8	0.19	2.39	75.9	1.74	2.30
SA-O1				Org	sandy loam	6.9	0.369	25.8	15.6	10.0	0.14	1.58	68.1	3.37	4.94
SA-O2				Org	sandy loam	6.7	0.319	37.3	31.0	15.9	0.20	2.87	114.6	5.44	4.75
SA-O3				Org	sandy loam	7.7	0.803	75.8	39.0	43.6	0.24	3.09	131.2	6.44	4.91
SA-O4				Org	clay	8.0	0.681	49.6	35.8	14.4	0.11	1.00	130.8	3.69	2.82

LAT, latitude; LON, longitude; MAT, mean annual temperature; MAP, mean annual precipitation; Org denotes organically managed soil, Conv denotes conventionally managed soil. EC, electrical conductivity salinity; WHC, water holding capacity; T-N, total nitrogen; OC, organic carbon; WSOC, water soluble organic carbon in soil water extract (soil∶water, 1∶1); MBC, microbial biomass carbon.

**Figure 4:**
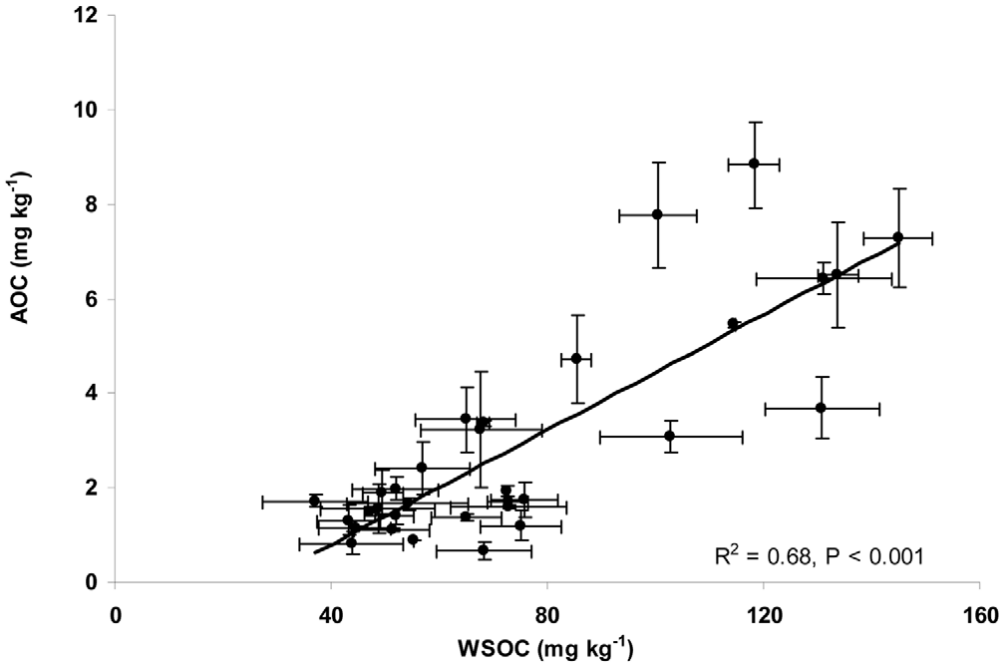
Linear regression analysis between assimilable organic carbon (AOC) and water soluble organic carbon (WSOC) in soil. The data represent the average of triplicate soil measurements.

**Figure 5:**
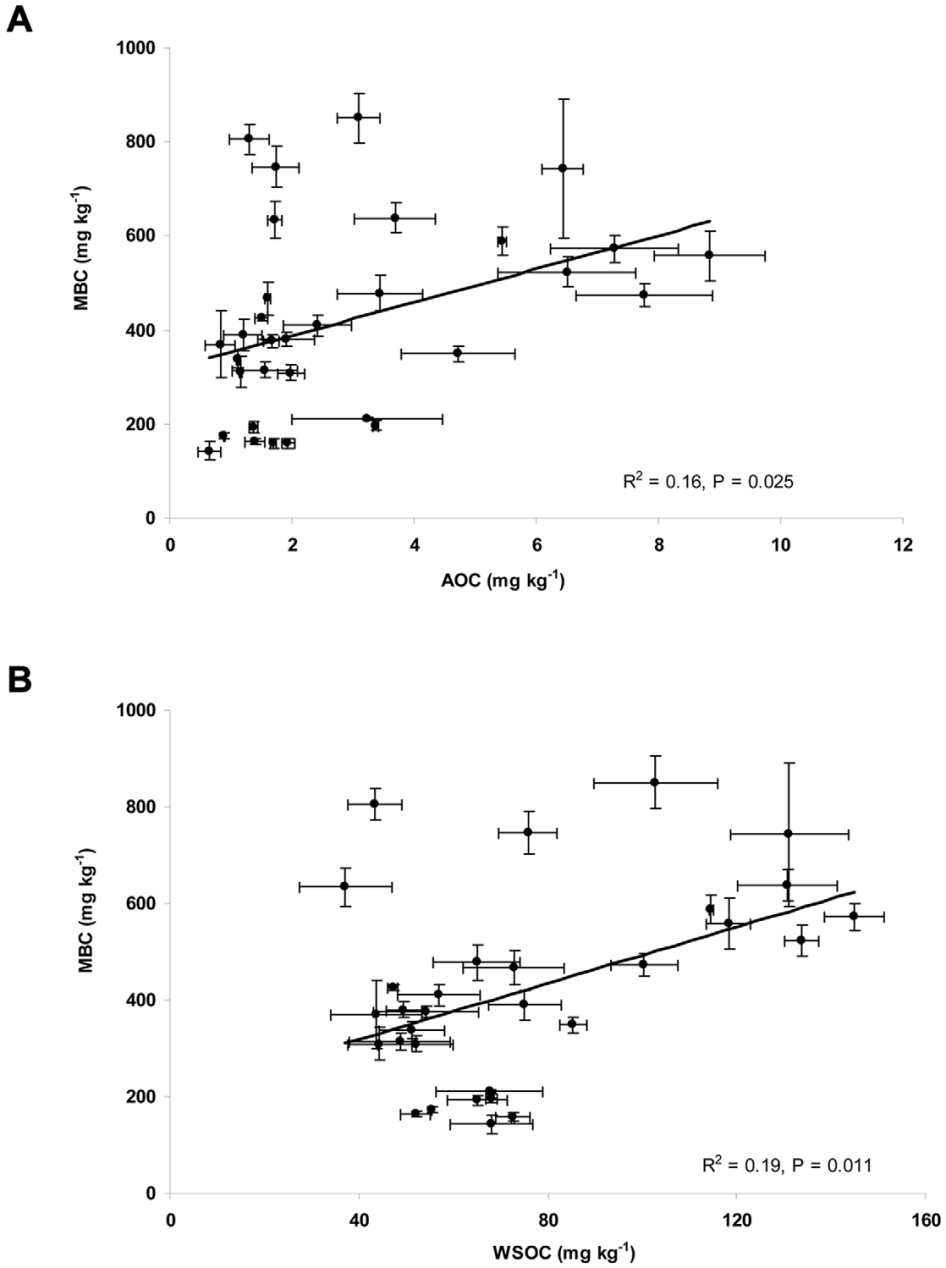
Correlation of soil microbial biomass as determined by microbial biomass carbon (MBC) with soil assimilable organic carbon (AOC) concentrations (5A) and with water soluble organic carbon (WSOC) concentrations (5B). The data represent the average of triplicate soil measurements.

Pearson correlation coefficients were calculated between soil properties and three soil organic carbon fractions, MBC, WSOC, and AOC. The results (Table 3) showed that AOC, WSOC, and MBC were positively correlated with MAP, WHC, pH, OC, and T-N, while the other factors including MAT, clay, and EC show different effects on those carbon fractions in soils tested. AOC was highly correlated with MAP of the soil sampling location, and WHC and EC of the soils. AOC was strongly correlated with water extractable organic carbon (WSOC) (*P*<0. 001, n = 32) (Fig. 4). The ratio of AOC to water soluble organic carbon (WSOC) was 3.60%, which is consistent with the range of values measured for AOC in water [8] which AOC comprises 0.1–10% of TOC. Also, the microbial biomass indicated by the concentration of microbial biomass carbon (MBC) was found to be significantly correlated with the AOC in soil water extracts (*P* = 0.025, n = 32) (Fig. 5A) and with WSOC in soil water extracts (*P* = 0.011, n = 32) (Fig. 5B), respectively.

**Table 3:** Pearson correlation coefficients between soil properties and MBC, WSOC, and AOC.

	Latitude	MAT	MAP	WHC	clay	pH	OC	T-N	EC
MBC	−0.127	+0.127	+0.127	+0.756***	+0.643***	+0.162	+0.608***	+0.476***	+0.207
WSOC	+0.115	−0.115	+0.373*	+0.253	−0.011	+0.189	+0.172	+0.120	−0.488***
AOC	−0.164	+0.164	+0.389*	+0.357*	+0.244	+0.122	+0.191	+0.260	−0.064

MAT, mean annual temperature (°C); MAP, mean annual precipitation (mm); WHC, water holding capacity (%); OC, organic carbon (%); T-N, total nitrogen (%); EC, electrical conductivity salinity (dS m^−1^); MBC, microbial biomass carbon (mg kg^−1^); WSOC, water soluble organic carbon (mg kg^−1^); AOC, assimilable organic carbon (mg kg^−1^) “+” indicates a positive correlation, “−” indicates a negative correlation,

* denotes significant at the 0.05 level,

*** denotes significant at the 0.001 level.

## Discussion

In this study, a naturally occurring luminous marine bacterium *V. harveyi* BB721 was used to determine AOC in soil water extracts. The bioluminescence reaction involves the oxidation of a long-chain aliphatic aldehyde and the reduction of flavin mononucleotide by an enzyme called luciferase coded by *lux* operon. The *luxCDABE* operon derived from *Vibrio* strains has been fused into microbial genomes, and the *lux* fusions have been used for gene expression studies. The *lux* gene has been good reporter gene to quantify carbon flow through components of the rhizosphere microbial community, as well as other nutrient fluxes [29]. Recently, *luxCDABE* has been integrated into the classical AOC test strains, P17 and NOX, and the resultant genetically modified strains have shown to be promising in AOC assays for water samples [22]. The biomass determined by peak bioluminescence intensity was comparable with that determined by plate counts [22]. The major advantage of the P17-lux and NOX-lux was a major reduction of the work load associated with the plate count, although the overall time required for AOC assay was not significantly decreased. The disadvantage of the use of the genetically modified strains is that the expression of luciferase genes was shown to be marginally effective in P17-*lux* whose bioluminescence signal was at least one magnitude lower than that of NOX-*lux* under the same conditions [22], [23]. Also, in order to produce the luminescence, aliphatic aldyde, e.g. decanol, was needed to stimulate the bioluminescence signal. Therefore, a simple, fast, and non-growth based AOC test developed in this study has reduced some of the problems encountered in the growth based assay described above. The use of naturally occurring luminous strain such as the one in this study has many advantages. *V. harveyi* naturally harbors the *lux* operon, which is believed to provide the best sensitivity and detection limit compared to other genetically modified AOC test strains [23], [24]. However, the use of pure cultures also have some disadvantages, those include limited substrate range and uneven response to different substrates. The fact that *V. harveyi* BB721 responded differently to various substrates was not likely caused by the uptake rates, but might be due to energy yield rates when cells metabolize a given amount of carbon in different organic carbon formats. Apparently, additional study might be needed to elucidate the mechanisms involved in low molecular weight organic carbon uptake, which in turn will help to improve the AOC assay development for soil samples. It has also been reported that *V. harveyi* produces bioluminescence constitutively, independent of cell density and the addition of autoinducer [26]. By using the AOC rapid assay, we have demonstrated that bioluminescence can be directly correlated to a class of assimilable organic substrates that can be rapidly metabolized in a water sample. In contrast to the standard growth based method, the rapid assay is designed to use a single strain of bioluminescent *V. harveyi* in combination with a luminometer to measure metabolic activity. It uses glucose-carbon to determine the response of bioluminescent *V. harveyi* to various known concentrations of glucose.

Many different factors must be evaluated to optimize procedures for measuring AOC in soil using a bioluminescence based assay system. Results of this study showed that the most important factors for successful application of this method included careful preconditioning of the cells used for the assay, calibration of the assay using an AOC standard series based on glucose, and development of precise protocols for carrying out the assay to obtain maximum sensitivity and range of detection. The assay was shown to provide accurate and consistent measurements of AOC from soils having different soil physical and chemical properties. We also show that AOC levels were strongly correlated with microbial biomass values for different soils, suggesting that AOC concentrations under steady state conditions (no recent organic amendments) are correlated with microbial biomass and activity.

Bioluminescence-based tests for measuring AOC in water have been in use for several years using genetically modified AOC test strains, P17 and NOX [22], [23]. A recent work [24] showed the feasibility of using the naturally occurring luminous strain, *V. harveyi* BB721, in AOC assays for saline water samples. The strength of luminescence signals of the microbial biomass yielded were significantly related to the amount of organic carbon added. However, like the other AOC tests developed previously, this new protocol involved long term incubation of cells in water samples. In contrast, the AOC test developed in this study was based on the assumption that luminescence intensity of starved bioluminescent cells is proportional to the concentration of glucose added in water and thus requires relatively short incubation times in comparison to assays that requires measurement of final cell densities after several days of cell growth. A previous study has shown that the emission of bioluminescence by strain BB721 can consume significant amounts of energy during the reaction catalyzed by luciferase [30]. The intensity of light was proportional to the amount of organic carbon inside the cell, and/or in the medium. Therefore, it is necessary to precondition the cells by using late log phase cells that are then starved for 30 min before being used for AOC tests (Fig. 3).

To test the methods developed here, the AOC concentrations from 32 soils with different chemical and physical properties were measured. Overall, soils collected from north California having a cooler climate contained higher AOC, while those in south California and Arizona with warmer climate had relatively lower AOC. The results correlated with annual mean temperature and precipitation, since high temperature and high precipitation generally result in a faster turnover of labile organic carbon. Differences in AOC quantities also were significantly and positively affected by total soil organic matter, total nitrogen, and humic acid concentrations.

Acetate is widely used as a reference carbon to quantify AOC in water samples. However, in the current study, glucose was the selected carbon source because it generated a luminescence signal greater than acetate and the other low molecular weight organic carbon substances. This result was confirmed in a recent study by Weinrich et al [24]. Comparison of hydrogen/carbon and oxygen/carbon molar ratios between WSOC and selected model compounds via van Krevelen diagram (a graphical-statistical method that cross-plots the hydrogen/carbon in function of the oxygen/carbon molar ratios of carbon compounds) [31] has shown that the WSOC composition is often positioned toward the carbohydrates region, indicating that carbohydrates are dominant components of WSOC [32]. A recent study [33] revealed that glucose was the major limiting substance for bacterial growth in soils. Both facts, to some extent, justify the use of glucose in the current study. Additional experiments are going on in our laboratory to investigate the feasibility of the classical water AOC test for soil water extract. WSOC in soil water extract is comprised of relatively labile compounds such as sugars, organic acids, and simple carbohydrates, and more complex substances such as branched carbohydrates, phenols, fulvic acids [34]. It should be noted that the use of single substrate in AOC assays might not well reflect the full picture of soil AOC. The results obtained in the current study might be able to reflect the actual level of AOC in soil water extract; however the results obtained at least determined the most bioavailable fraction of the total AOC in soil water extracts that has a direct correlation to the microbial biomass indicated MBC as discussed below.

The fraction of AOC contained in WSOC was shown to vary for different soils, but on the average, AOC comprised 3.60% of WSOC. This value is similar to the values that have been measured for the ratio of AOC to TOC in water samples. WSOC is only a small fraction of the total organic carbon pool in soils that is comprised of organic materials that are in different stages of decomposition. In soils, organic carbon molecules are important binding agents that cement soil particles into microaggregates. The microaggregates are protected until the soil aggregates are physically disrupted. Since WSOC is comprised of physically protected carbon as well as carbon contained in soil macropores accessible to microorganisms, AOC will likely vary for soils having different textures and aggregate stabilities. WSOC liberated by the extraction process can also be adsorbed on the solid phase of soil such that there will always be a small portion remaining in the soil samples after the extraction. The extraction efficiency of WSOC from soil depends on the volume of water used and means of extraction, soil properties, and the WSOC itself. Studies have shown that increasing the temperature of the extraction water results in higher yields of WSOC from soils and sediments [32].

One of the most interesting findings in the present study was that AOC concentrations in soil could be significantly correlated with the soil microbial biomass as measured by MBC. This is in agreement with the observation that there is also a strong correlation between WSOC in soil water extracts and MBC extracted from the same soils, indicating that the most bioavailable fraction of WSOC in soil water extract, largely AOC, might play an important role in microbial biomass under steady state. The findings also revealed that changes in soil environmental conditions effect the correlations between AOC levels and microbial biomass. Previous evidence showed that the biomass of soil microorganisms is usually limited by bioavailable organic carbon [35], and the ratio of microbial biomass carbon to total organic carbon typically varies from 2% to 4% [36]. It was found that the AOC concentrations were significantly correlated with MBC obtained by well established chloroform-fumigation-extraction protocol [37]. Microbial carbon has two different components. One is a stable, structural component comprising the cell wall, and the second is a more labile, largely cytoplasmic component, which play an important role in cell functioning [38]. It might be helpful to know the relationship between the AOC levels and the biomass calculated from the difference between the amount of CO_2_ evolved during incubation of fumigated and non-fumigated soil [39]. A similar relationship might be observed between respiration rates and AOC as observed between MBC and AOC.

### Conclusion

The research presented here shows that using *Vibrio harveyi* BB721, AOC can be readily estimated in a variety of different soil water extracts and that there is a direct correlation between soil AOC levels and microbial biomass carbon. This suggests that under steady state concentrations, AOC in soil water extracts are in equilibrium with the soil microbial biomass. The strong correlation (*P*<0.01) between AOC concentrations in soil water extracts with MBC revealed that the AOC in soil water extracts might be an important indicator of microbial biomass in soils. More importantly, the AOC assay developed in the current study is fast, simple, and may be easily automated when fully optimized. It could be used as a routine AOC test for monitoring soil and sediment quality and health. As the concentrations of labile carbon vary for different organic materials having different physical and chemical properties and their relative state of decomposition, methods to assay the AOC in soils provide a means to assess the ability of organic amendments to promote microbial growth and to track changes in growth potential over time. The AOC levels in soils tested in this study ranged from 0.66 to 8.83 mg kg^−1^. These significantly correlated with total soil organic carbon, total nitrogen. Soil AOC was highly correlated with water soluble organic carbon and the concentration of MBC, thereby providing a means to assess the potential for microbial growth in soils.
